# Upregulated osterix promotes invasion and bone metastasis and predicts for a poor prognosis in breast cancer

**DOI:** 10.1038/s41419-018-1269-3

**Published:** 2019-01-10

**Authors:** Bing Yao, Jue Wang, Shuang Qu, Yang Liu, Yuci Jin, Jianlei Lu, Qianyi Bao, Lingyun Li, Hongyan Yuan, Changyan Ma

**Affiliations:** 1 Jiangsu Key Laboratory of Xenotransplantation, Nanjing Medical University, Longmian Road 101, 211166 Nanjing, Jiangsu China; 2 Department of Medical Genetics, Nanjing Medical University, Longmian Road 101, 211166 Nanjing, Jiangsu China; 3 Division of Breast Surgery, the First Affiliated Hospital with Nanjing Medical University, 210029 Nanjing, China; 4 Department of Orthopedics, the First Affiliated Hospital with Nanjing Medical University, 210029 Nanjing, China; 5 Department of Oncology and Lombardi Comprehensive Cancer Center, Georgetown University Medical Center, Washington, DC 20007 USA

## Abstract

Approximately 70% of patients with advanced breast cancer develop bone metastases, accompanied by complications, such as bone pain, fracture, and hypercalcemia. However, our understanding of the molecular mechanisms that govern this process remains fragmentary. Osterix (Osx) is a zinc finger-containing transcription factor essential for osteoblast differentiation and bone formation. Here, we identified the functional roles of Osx in facilitating breast cancer invasion and bone metastasis. Osx upregulation was associated with lymph node metastasis and was negatively prognostic for overall survival. Knockdown of Osx inhibited invasion of breast cancer and osteolytic metastasis by downregulating MMP9, MMP13, VEGF, IL-8, and PTHrP, which are involved in invasion, angiogenesis, and osteolysis; overexpression of Osx had the opposite effect. Moreover, MMP9 was a direct target of Osx and mediated the Osx-driven invasion of breast cancer cells. Together, our data showed that Osx facilitates bone metastasis of breast cancer by upregulating the expression of a cohort of genes that contribute to steps in the metastatic cascade. These findings suggest that Osx is an attractive target for the control of bone metastasis of breast cancers.

## Abstract (web summary)

Osx facilitates bone metastasis of breast cancer by upregulating the expression of a cohort of genes that contribute to steps in the metastatic cascade. These genes include MMP9, VEGF, as well as IL-8, PTHrP, and MMP13. MMP9 is a newly identified target of Osx.

.

## Graphical abstract

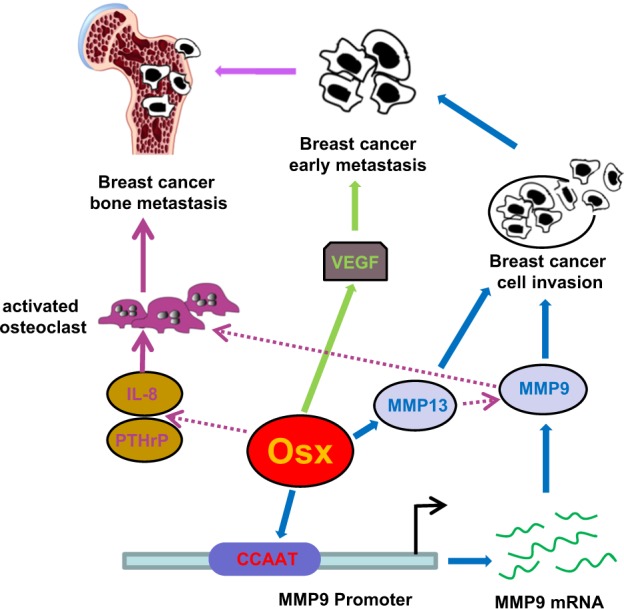

## Introduction

Breast cancer, the second most commonly diagnosed cancer worldwide, affects ~12% of women and causes 14% of all cancer-related fatalities^1^. Metastases cause more than 90% of deaths of breast cancer patients^2^, and ~70% of patients with metastatic breast cancer develop bone metastases during the course of their disease^3,4^. Bone metastases impair the quality of life due to hypercalcemia, bone pain, fractures, nerve compression, and a reduction in mobility and social function^5,6^. Therefore, control of bone metastases in breast cancer is a problem for clinical practice.

Cancer metastasis is a complex, multistage process that includes the steps of local invasion, intravasation, survival in the circulation, extravasation, and colonization^7^. Involved in this process are various molecules, including: (1) metastasis initiation molecules, such as TWIST1, matrix metalloproteinases (MMPs), HIF1A, and vascular endothelial growth factor (VEGF)^8–10^; (2) metastasis progression molecules, such as PTGS2, EREG, LOX, ANGPLTL4, and CLDN2^11–14^; and (3) metastasis virulence molecules, such as interleukin 8 (IL-8), vascular cell adhesion molecule 1 (VCAM-1), and parathyroid hormone-related protein (PTHrP)^15–17^. Among these molecules, IL-8, VCAM-1, and PTHrP are factors involved in bone metastasis. Despite progress with respect to the understanding of the molecular basis of bone metastasis in breast cancer, knowledge of the mechanisms underlying this process must be extended to identify targets for the prevention and treatment of bone metastases.

Osterix (Osx/SP7), a member of the SP family of zinc finger-containing transcription factors, is a master regulator of osteoblast differentiation and bone formation^18^. Osx regulates the differentiation of preosteoblasts into mature osteoblasts in a step downstream of Runx2. Deletion of Osx in mouse embryos results in the absence of osteoblasts and no bone formation^18,19^. By binding to specific GC-rich sequences, Osx regulates the expression of osteogenic factors, including osteonectin, osteopontin, osteocalcin, and alkaline phosphatase^20,21^. Further, in osteoblasts, VEGF and MMP13 are target genes of Osx^22–24^. A microarray analysis has compared MMP gene expression profiles between osteoblasts from Osx wild-type mice and those from Osx knockout mice^24^. In osteoblasts from wild-type mice, MMP9 is the second most highly expressed member of the MMP family. Given the roles of VEGF in angiogenesis, MMP13 in osteoclast differentiation and activation^25^, and MMP9 in invasion, we postulated that Osx may promote bone metastasis in breast cancer by regulating proteins involved in the metastatic cascade.

In the present study, we clarified the roles of Osx in breast cancer invasion and bone metastasis and characterized the underlying mechanisms. For patients with breast cancer, Osx upregulation was associated with lymph node metastasis and was negatively prognostic for overall survival. By downregulating MMP9, MMP13, VEGF, IL-8, and PTHrP, knockdown of Osx inhibited invasion of breast cancer cells and osteolytic metastasis; overexpression of Osx had the opposite effect. Moreover, MMP9 was a target of Osx and mediated the Osx-driven invasion capacity of breast cancer cells. Thus, Osx promotes bone metastasis of breast cancer by upregulating proteins in the early and late stages of the metastatic cascade. These findings suggest that Osx is an attractive target for the control of bone metastasis of breast cancers.

## Results

### High Osx expression is associated with lymph node metastasis and a poor prognosis in breast cancer

**Fig. 1:**
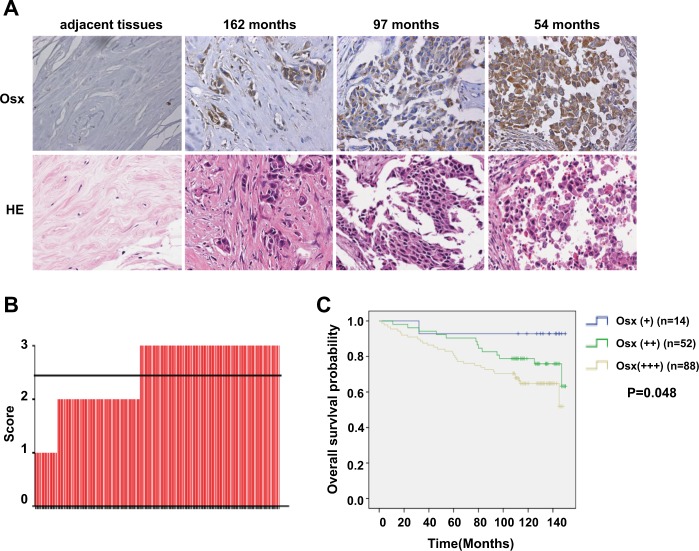
Osx expression is associated with survival. **a** Osx expression correlates with overall survival time, based on a microarray of breast cancer tissues. Representative samples of a breast cancer tissue microarray with different overall survival times and adjacent tissues stained for Osx are shown. Magnification, ×400. **b** Overall expression of Osx in 154 cases from the breast cancer tissue microarray: 1. low expression; 2. moderate expression; and 3. high expression. **c** Osx expression levels negatively correlate with overall survival. Univariate analysis showed that patients with higher Osx expression had a shorter survival time than patients with lower Osx expression (*P* = 0.048)

To investigate the role of Osx in metastasis of breast cancer, the expression of Osx was detected by use of immunohistochemistry in a tissue microarray of 154 breast cancers. Clinicopathologic analysis showed that Osx levels were elevated in tumors of patients with short survival and that Osx was not expressed in adjacent tissues (Fig. 1a). Osx was highly expressed in 57.1% of the breast cancer tissues (score: 3), but was weakly expressed in only 9.1% of the breast cancer tissues (score: 1) (Fig. 1b). Patients with higher Osx expression had a poorer survival rate than patients with lower Osx expression (Fig. 1c). Osx expression was significantly correlated with lymph node metastasis, whereas there was no statistical significance between Osx expression and patients’ age, tumor size, TNM stage, or histological sub-type (ER, PR, AR, HER2 status), nor was there an association between Osx expression and Ki-67, EGFR, P53, or CK56 expression (Table 1). Together, these data showed that, for breast cancer, high Osx expression was associated with a poor prognosis and with lymph node metastasis, suggesting that Osx is involved in breast cancer metastasis.

**Table 1:** Correlations between Osx expression and clinicopathologic factors in tissue samples of invasive ductal carcinoma

Variable	Osx			*P*-value
	+	++	+++	
Age				0.208
≤50 years	8	26	39	
>50 years	4	24	53	
Tumor size				0.792
≤2 cm	2	13	23	
>2 cm	10	37	69	
Lymphnode involvement				0.040
Negative	6	21	35	
Positive	6	29	57	
ER status				0.786
Negative	3	15	31	
Positive	9	35	61	
PR status				0.414
Negative	4	17	41	
Positive	8	33	51	
AR status				0.490
Negative	11	47	77	
Positive	3	5	11	
HER2 status				0.796
Negative	9	33	60	
Positive	3	17	32	
Clinical tumor stages				0.313
I	5	17	22	
II	9	35	61	
III	0	0	5	
TNM stages				0.758
I	1	5	10	
II	9	34	48	
III	4	13	30	
Ki-67				0.614
Negative	1	1	3	
Positive	13	51	85	
EGFR				0.398
Negative	2	13	27	
Positive	12	39	61	
P53				0.079
Negative	6	8	18	
Positive	8	44	70	
CK56				0.353
Negative	13	47	73	
Positive	1	5	15	

### Osx promotes invasiveness of breast cancer cells

**Fig. 2:**
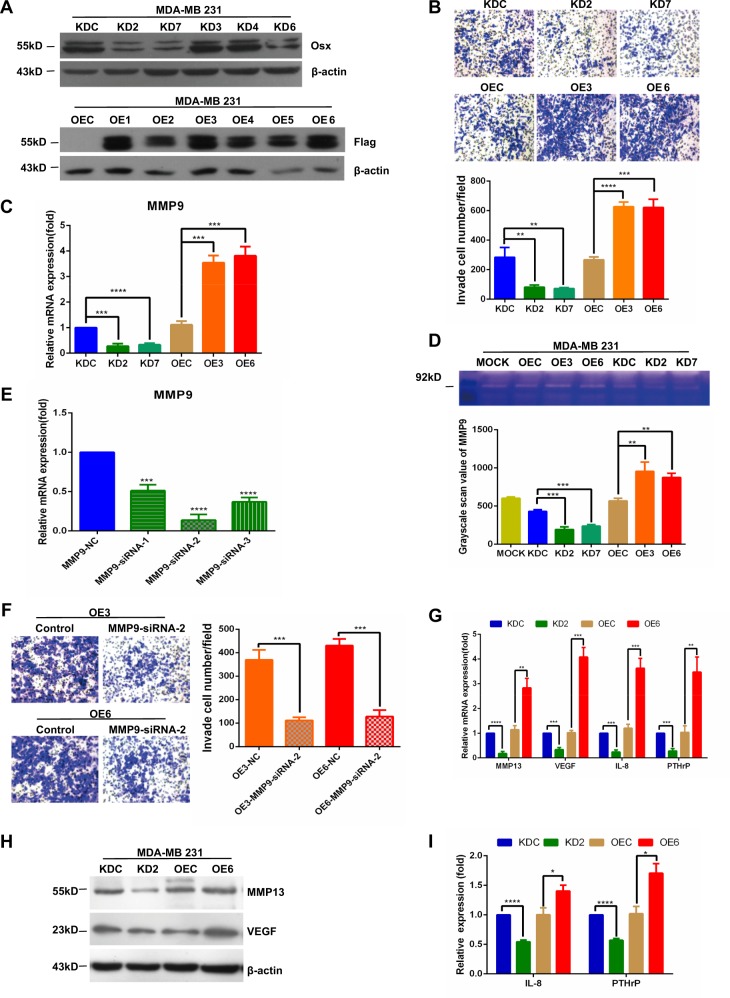
Osx promotes the invasiveness of  breast cancer cells. **a** Identification of cell lines with stable Osx overexpression or knockdown. MDA-MB 231 cells were infected with lentiviruses containing Osx cDNA, Osx-specific siRNA, or the corresponding control virus. The cells were treated with puromycin at 48 h after transfection, and stable clones were subsequently selected. Stable expression was detected by Western blot analyses with anti-Osx or anti-Flag antibodies. β-Actin was used as an internal control. **b** Effects of Osx on the invasiveness of breast cancer cells. The cells were seeded into Matrigel-coated invasion chambers and incubated for 24 h. The numbers of migrated cells were quantified by counting the numbers of cells in entire fields at ×200 magnification. Representative images (upper panel) and quantitative analyses of the invaded cells (lower panel) are shown. **c** MMP9 mRNA expression was measured by qRT-PCR in stably transfected cells. β-Actin was used as an internal control. **d** MMP9 enzymatic activity in stably transfected cells was measured by gelatin zymography. Representative images of gelatin zymography (upper panel) and quantitative analyses of the enzymatic activity (lower panel) are shown. **e** MMP9 siRNA and Scr siRNA were transfected into MDA-MB 231 cells. qRT-PCR was performed at 24 h after transfection to measure MMP9 mRNA levels. β-Actin was used as an internal control. **f** Cells with stable Osx overexpression were transiently transfected with MMP9 siRNA or Scr siRNA, and then Transwell assays were performed to determine if MMP9 is required for Osx-mediated invasion. Magnification, ×200. The numbers of cells that passed through the Matrigel were counted under a microscope in entire fields. Representative images (left panel) and quantitative analyses of the invaded cells (right panel) are shown. **g** Effects of Osx on the mRNA expression of MMP13, VEGF, IL-8, and PTHrP were examined by qRT-PCR using cDNA from stably transfected cells. β-Actin was used as an internal control. **h** MMP13 and VEGF protein expression in stably transfected cell lines was detected by Western blot analysis. β-Actin was used as an internal control. **i** IL-8 and PTHrP protein levels in culture media from stably transfected cells were determined by ELISA. Values are presented as the mean ± SD. * Indicates *P* < 0.05, ** indicates *P* < 0.01, *** indicates *P* < 0.001

To determine the role of Osx in the development of metastasis, we assessed the effect of Osx on tumor cell invasion, a characteristic of metastatic capacity. MDA-MB 231 cell lines with stable Osx knockdown or overexpression and corresponding control cell lines were established by lentivirus infection (Fig. 2a). Transwell assays showed that knockdown of Osx inhibited the invasiveness of MDA-MB 231 cells, whereas overexpression of Osx promoted their invasiveness (Fig. 2b).

Since MMP9, a gelatinase involved in tumor invasion, exhibits the second largest expression difference (a 15.28-fold difference) among the MMP family members in osteoblasts between Osx-knockout mice and Osx-wild-type mice^24^, we hypothesized that Osx promotes invasiveness of breast cancer cells by upregulating MMP9 expression. To test this hypothesis, we performed qRT-PCR and gelatin zymography assays using MDA-MB 231 cells. Knockdown of Osx decreased MMP9 mRNA expression and enzymatic activity, whereas overexpression of Osx showed the opposite effect (Fig. 2c, d). Next, scrambled (Scr) siRNA or MMP9 siRNA was transfected into MDA-MB 231 cells with stable Osx overexpression, and their capacity for invasion was evaluated. All three RNA interference targets decreased MMP9 mRNA levels; target 2 (siRNA-2) displayed the highest interference efficiency (Fig. 2e). When siRNA-2 was transiently transfected into MDA-MB 231 cells with stable Osx overexpression, the effect of Osx on cell invasion capacity was impaired (Fig. 2f). These results indicated that Osx promotes invasiveness, at least in part by upregulating MMP9 expression.

Metastasis is a multistep process requiring the participation of various factors, and bone is the primary tissue to which breast cancer metastasizes. Thus, we next assessed the effect of Osx on the expression of angiogenesis regulator, VEGF, as well as the osteolytic factors MMP13, IL-8, and PTHrP. Knockdown of Osx decreased mRNA expression of VEGF, MMP13, IL-8, and PTHrP (Fig. 2g). In contrast, overexpression of Osx upregulated mRNA expression of these factors (Fig. 2g). In agreement with these findings, protein expression of MMP13 and VEGF was low in Osx-knockdown cells and high in cells overexpressing Osx (Fig. 2h). The downregulation of IL-8 and PTHrP caused by Osx knockdown and the upregulation of IL-8 and PTHrP caused by Osx overexpression were verified by ELISA (Fig. 2i). Similar results were observed in another triple negative breast cancer cell line HCC 1937 (Supplementary Fig. 1).

### MMP9 is a target of Osx

**Fig. 3:**
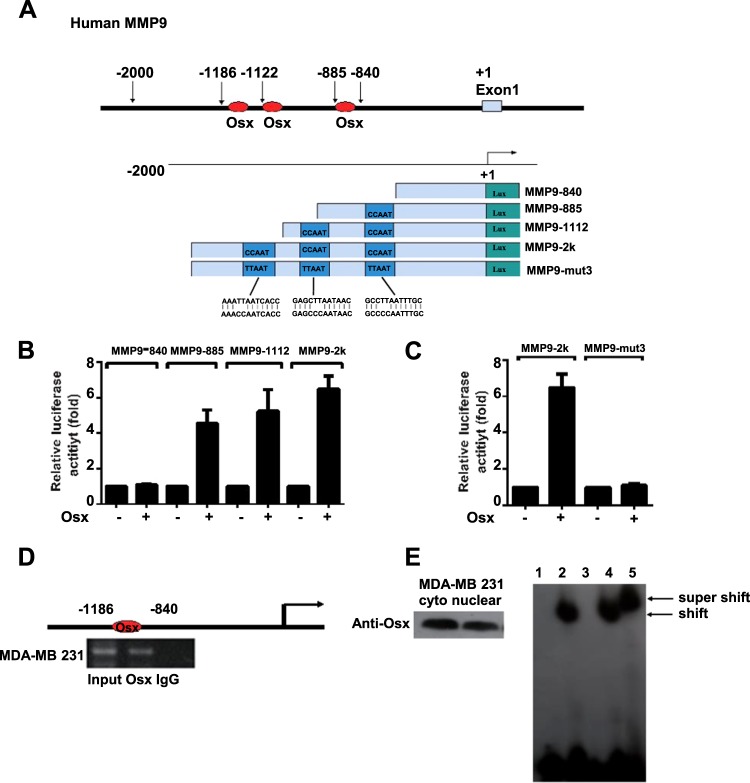
MMP9 is a direct target of Osx. **a** The promoter region (−2.0 kb) of human MMP9, with Osx-binding elements at positions −885 (CCAAT), −1122 (CCAAT), and −1186 (CCAAT) (upper panel). Schematic representation of the Osx-deletion mutants (lower panel). **b** and **c** Identification of the Osx-binding site in the promoter of the MMP9 gene. HEK 293T cells were transfected with the MMP9 promoter-luciferase reporter and β-gal construct without or with Flag-tagged Osx-expressing plasmids using GBfectene Elite transfection reagent. Luciferase activity was measured at 36 h after transfection and normalized to β-gal activity. Values are presented as means ± SD. **d** Endogenous Osx in MDA-MB 231 cells is associated with the native MMP9 promoter. ChIP assays were conducted with MDA-MB 231 cells as well as anti-Osx antibody. IgG was used as a negative control. The precipitated chromatin was analyzed by PCR. **e** Osx binds to MMP9 promoter oligonucleotides. The MMP9 DNA oligonucleotides were labeled with ^32^P. Nuclear extracts from MDA-MB 231 cells were incubated with the ^32^P-labeled DNA probe. The protein–DNA complexes were separated on 5% polyacrylamide gels, and the resulting signals were recorded on X-ray film. Lane 1: without nuclear extracts; lane 2: with the ^32^P-labeled DNA probe; lane 3: with the ^32^P-labeled DNA probe and excess unlabeled MMP9 promoter oligonucleotides; lane 4: with the mutant probe (CCAAT to TTAAT); lane 5: with anti-Osx polyclonal antibody

Osx belongs to the SP/KLF family of transcription factors, which bind to GC-rich sequences in the promoters of their target genes to regulate gene expression^18^. Osx upregulates osteocalcin promoter activity by binding to the CCAAT sequence, which is different from the canonical GC-rich Sp1 binding site^26^. To elucidate the mechanism by which Osx upregulates MMP9 mRNA expression, the promoter sequence of MMP9 was assessed by bioinformatics analysis. The MMP9 promoter had no canonical GC-rich Sp1 binding site; however, the promoter contained three CCAAT sequences (Fig. 3a). To define the precise Osx regulatory region in the MMP9 promoter, a series of truncated and mutated constructs of the MMP9 promoter reporter were generated. Luciferase reporter assays of the constructs showed that Osx activated the promoter reporters of MMP9-885, MMP9-1112, and MMP9-2k, but not that for MMP9-840 (Fig. 3b). Increases in the number of potential binding sites serially enhanced MMP9-885, MMP9-1112, and MMP9-2k activation (Fig. 3b). These data indicated that the promoter regions of MMP9-885, MMP9-1112, and MMP9-2k contain binding sites for Osx. Additionally, Osx-mediated MMP9 reporter activation was eliminated when the potential binding sites were mutated (Fig. 3c). In sum, these data show that the CCAAT sequence functions as an Osx-response element that facilitates Osx-mediated MMP9 promoter activation.

To determine whether endogenous Osx binds to the native Osx promoter, ChIP assays were performed for MDA-MB 231 cells. As shown in Fig. 3d, Osx was recruited to the MMP9 promoter. To determine if Osx binds to the MMP9 promoter directly, gel-shift assays were accomplished. As shown in lane 2 of Fig. 3e, Osx bound to MMP9 promoter oligonucleotides. Binding specificity was demonstrated by competition analyses involving an unlabeled wild-type probe, which abolished formation of the DNA–protein complex (lane 3). In contrast, the mutant probe (CCAAT to TTAAT) failed to compete for binding (lane 4). A supershift assay using a polyclonal antibody against Osx confirmed the presence of Osx in the DNA–protein complex (lane 5). Collectively, these analyses demonstrated that Osx activates MMP9 gene transcription by binding to the CCAAT regulatory element.

### Osx promotes local invasiveness of breast cancer cells in mice

**Fig. 4:**
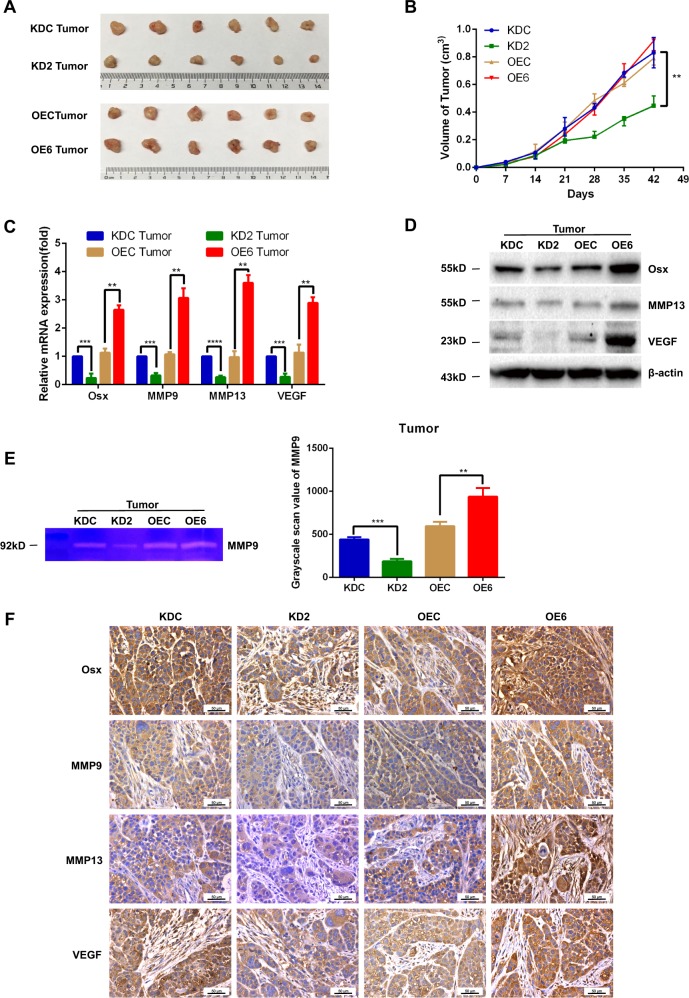
Osx promotes local invasion of breast cancer in mice. Stably transfected cells (2.5 × 10^6^) were injected into the mammary fat pads of 4-week-old female Balb/c nude mice. **a** Six weeks post-injection, the tumors were removed from the mice and imaged. **b** Mammary tumor growth was measured at the indicated times (six per group). **c** mRNA expression of Osx, MMP9, MMP13, and VEGF was examined by qRT-PCR in tumor tissues from each experimental group. β-Actin was used as an internal control. **d** Osx, MMP13, and VEGF protein expression in tumor tissues from each experimental group was examined by Western blot analyses. β-Actin was used as an internal control. **e** MMP9 enzymatic activity was determined by tissue zymography in tumor tissues from each experimental group. Representative images of gelatin zymography (left panel) and quantitative analysis of the enzymatic activity (right panel) are shown. **f** Expressions of Osx, MMP9, MMP13, and VEGF were examined by immunohistochemistry in tumor tissues from each experimental group. ** Indicates *P* < 0.01 and *** indicates *P* < 0.001

To explore the role of Osx in breast cancer invasiveness in mice, we injected the same amounts of MDA-MB 231 stable cells with Osx-knockdown or Osx-overexpression, as well as the corresponding control cells into separate groups of female nude mice to establish mammary fat pad xenografts. The Osx-knockdown cancer cells did not invade the surrounding stroma, whereas cancer cells of Osx xenografts invaded the surrounding stroma (Fig. 4a). Knockdown of Osx led to a delay in tumor growth, whereas overexpression of Osx had no appreciable effect on tumor growth (Fig. 4b). qRT-PCR analysis revealed that MMP9, MMP13, and VEGF mRNA levels were decreased in the Osx-knockdown xenografts, whereas overexpression of Osx increased the mRNA expression of these genes in the xenografts (Fig. 4c). The data pertaining to VEGF and MMP13 expression, as determined by Western blot analysis and by MMP9 enzymatic activity, measured by tissue gelatin zymography, were consistent with the mRNA expression levels (Fig. 4d, e). In agreement with the mRNA and protein expression results, the immunohistochemistry results showed that expression of MMP9, MMP13, and VEGF was lower in the tumors of Osx-knockdown xenografts and that overexpression of Osx increased the expression levels of these genes in the Osx-overexpressing xenografts (Fig. 4f). Collectively, these data showed that knockdown of Osx inhibited breast cancer invasiveness, whereas elevated Osx expression promoted invasiveness.

### Osx promotes osteolytic lesions

**Fig. 5:**
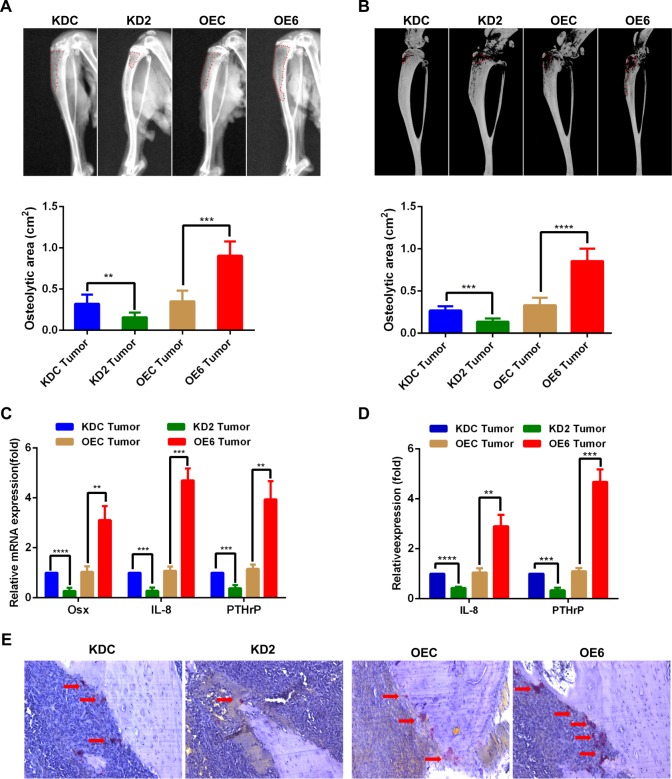
Osx promotes osteolytic lesions in mice. Stably transfected cells (1 × 10^5^) were injected into the tibiae of 4-week-old female Balb/c nude mice. The resulting bone lesions were examined at 8 weeks post-injection. **a** and **b** X-ray images (left panel) and microCT images (right panel) of bone lesions in representative mice from each experimental group (upper panel), as well as quantitative analyses (lower panel) are shown. **c** mRNA expression of Osx, IL-8, and PTHrP was examined by qRT-PCR in intratibial tumors in each experimental group. β-Actin was used as an internal control. **d** IL-8 and PTHrP protein levels were examined by ELISA in intratibial tumors in each experimental group. **e** TRAP staining of bone metastasis in each experimental group. The arrows indicate TRAP+ osteoclasts. A schematic model depicting the molecular mechanisms underlying Osx-mediated bone metastasis of breast cancers. Elevated Osx expression in breast cancer cells stimulates the expression of MMP9 and MMP13, which are responsible for the invasiveness of breast cancer cells. Osx also stimulates the expression of VEGF, a promoter of angiogenesis. Furthermore, Osx facilitates the expression of IL-8 and PTHrP, thus leading to the activation of osteoclasts and enhancing osteoclastogenesis. MMP13 is also involved in stimulating osteoclast differentiation and activation. By enhancing invasion, angiogenesis, and osteoclast activity, Osx participates in the early and late stages of bone metastasis. ** Indicates *P* < 0.01; *** indicates *P* < 0.001

To investigate the role of Osx in the development of osteolytic bone lesions, the same amounts of MDA-MB 231 cells with stable Osx-knockdown or Osx-overexpression, as well as the corresponding control cells were placed into the tibiae of nude mice. X-ray examination of the bones showed that osteolysis was low after implantation of Osx knockdown cells, whereas osteolysis was enhanced after implantation of Osx overexpressing cells (Fig. 5a). These results were consistent with the micro-CT results (Fig. 5b). Moreover, there was low IL-8 and PTHrP expression in the tumors with Osx-knockdown cells, whereas there was high IL-8 and PTHrP expression in the tumors with Osx-overexpressing cells, as demonstrated by qRT-PCR analysis and ELISA (Fig. 5c, d). Weaker osteoclast activity was evident at the bone–tumor interface in tumors with Osx-knockdown cells (arrows), whereas TRAP^+^ osteoclasts were enriched in bone metastases containing cells overexpressing Osx (Fig. 5e).

## Discussion

Metastatic breast cancer has imposed a challenge for clinicians, and there is an urgent need to search for and identify therapeutically relevant molecular targets specific to this type of disease. In the present study, we found that high Osx expression was associated with lymph node metastasis and a poor prognosis for breast cancer. Knockdown of Osx inhibited the invasive capacity of breast cancer cells and osteolytic metastasis by downregulating MMP9, MMP13, VEGF, IL-8, and PTHrP, whereas overexpression of Osx had the opposite effect. Moreover, MMP9 was a direct target of Osx and mediated the Osx-driven invasion potential of breast cancer cells. These findings support the involvement of Osx in bone metastasis of breast cancer.

Until several years ago, Osx expression was thought to be restricted to osteocytes, and previous studies focused on defining the functions of Osx in osteoblasts. In 2005, Cao et al. reported that Osx mediates antitumor activity in murine osteosarcomas^27^. However, the roles of Osx in breast cancer and the mechanisms underlying its effects in the disease remain unclear. In the present study, we found that Osx was highly expressed in breast cancer cells with higher metastatic potential. High Osx expression was associated with lymph node metastasis and a poor prognosis for breast cancer. These results suggest that, in breast cancer, Osx is involved as an oncogene.

To metastasize, cancer cells must initially escape from the complex physical barriers (extracellular matrix (ECM), basement membrane, and vasculature) located at the site of the primary tumor and then invade adjacent tissues. Invasiveness is a feature in the development of metastases. The MMPs are a family of structurally related, zinc-dependent endopeptidases capable of degrading components of the ECM^28^. The MMP family consists of four types of enzymes, including collagenases, gelatinases, stromelysins, and membrane-type MMPs^29^. High levels of MMP9, a gelatinase, are present in numerous cancers, including breast cancers^30^. MMP9 activity is regulated by transcriptional activation, enzymatic activation, or inhibition by tissue inhibitors of MMPs (TIMPs)^31^. Of these mechanisms, transcriptional activation of MMP9 appears to be the most complex and decisive manner by which MMP9 expression is regulated^32^. Through luciferase reporter assays, ChIP assays, and EMSA, we confirmed that Osx binds to the MMP9 promoter and consequently upregulates transcription of the MMP9 gene, thus enhancing MMP9 enzymatic activity. Furthermore, through RNA interference, we demonstrated that MMP9 mediates the promotion of invasion by Osx. Another collagenase, MMP13, which is implicated in ECM degradation^33^, highly expressed in breast cancer tissues, indicating that it is involved in breast cancer invasion and metastasis^34^. In osteoblasts, MMP13 is a target of Osx^24^. Consistent with this, we found that, in breast cancer cells, both MMP13 and MMP9 were upregulated by Osx, suggesting that Osx has a function in invasion, an initial stage in the metastasis cascade.

Angiogenesis is another step in cancer metastasis. The level of angiogenic activity in breast cancer is a determinant of disease progression and survival^35^. VEGF is responsible for regulating angiogenesis, and blocking the action of VEGF is a promising anti-angiogenic strategy for treating various types of solid tumors, including breast cancers. In osteoblasts, Osx upregulates VEGF expression by binding to its promoter^22^, suggesting that Osx participates in angiogenesis. We demonstrated that, in breast cancer cells, VEGF mRNA and protein expressions were upregulated by Osx, indicating that Osx is involved in regulation of breast cancer angiogenesis, and we found that Osx promotes breast cancer angiogenesis through upregulation of S100A4 and VEGF (data not shown).

Breast cancer metastasizes to bone in more than 70% of patients with late-stage cancer^36^. Bone metastases can be classified as osteolytic, osteosclerotic, or mixed (both features coexist), according to their histological and clinical features^37^. Osteolytic metastases are the most common metastases in breast cancer, although mixed and osteoblastic metastases occur in a significant number of patients^38^. Tumor–bone molecular interactions mediated by various factors drive a cycle that perpetuates skeletal metastases^39^. Breast cancer cells produce various osteolytic mediators, of which IL-8 and PTHrP cause bone destruction by inducing the differentiation and activation of osteoclasts^40^. Our data showed that expressions of both IL-8 and PTHrP were increased by Osx overexpression, thus enhancing osteoclast activity. In addition, in bone metastasis, MMP13 is involved in stimulating osteoclast differentiation and activation^25,41^. Therefore, elevated Osx expression in breast cancer cells promotes the expression of osteoclast activators, raises osteoclast activity, and promotes the occurrence of osteolytic lesions.

In conclusion, the present study showed that Osx promotes bone metastasis of breast cancers by: (1) upregulating MMP9, a molecule associated with invasion; (2) upregulating VEGF, a regulator for angiogenesis; and (3) upregulating IL-8, PTHrP, and MMP13, inducers of osteoclast activity (Fig. 5f). Moreover, we identified MMP9 as a target of Osx. These results suggest that Osx is an attractive target for the control of bone metastasis of breast cancers.

## Materials and methods

### Cell culture and reagents

Human embryonic kidney HEK 293T cells were maintained in high-glucose Dulbecco’s modified Eagle medium (DMEM), and human breast cancer MDA-MB-231 cells were maintained in Roswell Park Memorial Institute-1640 medium (RPMI-1640). The media were supplemented with 10% fetal bovine serum (FBS), 100 units/ml penicillin, and 100 μg/ml streptomycin. All the cells were purchased from the ATCC (Manassas, VA, USA) and cultured at 37 °C in a humidified atmosphere containing 5% CO_2_. Lipofectamine 2000 was obtained from Invitrogen (Carlsbad, CA, USA), and GBfectene Elite Transfection Reagent was obtained from Genebank Biosciences Inc. (Suzhou, China). Puromycin was purchased from Sigma (Sigma-Aldrich, USA) and FastStart Universal SYBR Green Master Mix (Rox) from Roche (Roche Diagnostics, Indianapolis, IN, USA). RNAiso plus and PrimeScript RT Reagent Kits were purchased from TaKaRa Biotechnology (Dalian, China). Matrigel was purchased from BD Biosciences (New Jersey, USA).

### Tissue microarray

The microarray of breast cancer tissue (HBre-Duc170Sur-01), which contained 154 samples of invasive ductal carcinomas from patients aged 29–82 years (average age: 55 years), was purchased from Outdo Biotech Co., Ltd. (Shanghai, China). Ten samples had paired samples of adjacent normal breast tissue. The patients whose samples were included in the microarray underwent surgery from January 2001 to August 2004. All patients were followed up for a median of 35 months until July 2014. All pathologic diagnoses were included with the product manufacturer’s instructions. The stained tissue microarray samples were assessed and scored with a semiquantitative scale by two independent blinded investigators.

### Plasmids, lentiviruses, and stable cell lines

Human MMP9 promoter constructs spanning −840/+1, −1100/+1, −1174/+1, and −2006/+1 bp containing zero, one, two, or three CCAAT sites, respectively, were generated by PCR amplification with the following PCR primers: Osx (−840/+1; forward), 5′-CCCTCGAGTTCTAAACATTTTATATG-3′; Osx (−1100/+1; forward), 5′-CCCTCGAGCCTGGTTTGGTGATTCCAAG-3′; Osx (−1174/+1; forward), 5′-CCCTCGAGGCAGTTGAAGAATCCTAAG-3′; Osx (−2000/+1; forward), 5′-CCCTCGAGACGGTGCTTGACACAGTAA-3′; and a reverse primer, the sequence of which was specific to all three Osx promoter constructs: 5′-CCAAGCTTGGTGAGGGCAGAGGTGTCTG-3′. The underlined letters indicate the Xho I and Hind III sites on the forward and reverse primers, respectively. The PCR products were inserted into the pGL3 vector. CCAAT mutations were introduced into the MMP9 promoter construct with overlap PCR with the following primers: MMP9-mut1: 5′-TATCCTGCCTTAATTTGCAGTTGAAGAA-3′; MMP9-mut2: 5′-TAGAGCTTAATAACCTGGTTTGGT-3′; and MMP9-mut3: 5′-AAATTAATCACCACCATCC-3′. All constructs were verified by DNA sequencing. The flag-tagged Osx expression plasmid was constructed as previously described^39^. The lentivirus containing Osx cDNA (GenBank accession No. NM_003620.3) was constructed by SOB Medical Biotechnology Co., Ltd. (Shanghai, China). The lentivirus containing Osx-specific small interfering RNA (siRNA) (5′-AAGCACTAATGGGCTCCTT-3′) was constructed by Shanghai Genechem Co., Ltd. (Shanghai, China). The indicated non-silencing (NS) sequence (5′-CGTACGCGGAATACTTCGA-3′) was used as a control. MDA-MB 231 cells were infected with lentiviruses. The cells were exposed to puromycin at 48 h post-transfection, and clones with stable expression were subsequently selected.

### Cell invasion assay

Briefly, cells were seeded in Matrigel (BD Biosciences, USA)-coated inserts in 24-well tissue culture plates. After incubation for 24 h, the cells that had migrated to the lower side of the inserts were stained with 1% crystal violet and counted under a microscope.

### Luciferase assay

HEK 293T cells were co-transfected with the MMP9 promoter-luciferase reporter, β-gal construct, and Flag-tagged Osx expression plasmids by using GBfectene Elite Transfection Reagent, according to the manufacturer’s instructions. At 36 h after transfection, the cells were lysed with 1 × reporter lysis buffer, and luciferase activity was measured with a Luciferase Reporter System (Promega Corp., Madison, USA) by using a GloMax™ Base Instrument (Promega). Relative luciferase activity was normalized to β-gal activity to determine transfection efficiency.

### Electrophoretic gel mobility shift assay (EMSA)

The following wild-type and mutated probes were used in the experiment: MMP9-880 (forward: 5′-GTAATTAAAACCAATCACCACCATCCGTTG-3′ and reverse: 5′-CAACGGATGGTGGTGATTGGTTTTAATTAC*-*3′) and MMP9-880 mutant (forward: 5′-GTAATTAAAATTAATCACCACCATCCGTTG-3′ and reverse: 5′-CAACGGATGGTGGTGATTAATTTTAATTAC-3′).

### Chromatin immunoprecipitation (ChIP) assays

ChIP assays were accomplished as described previously^40^. Briefly, formaldehyde cross-linking was performed in MDA-MB 231 cells, and the resulting samples were sonicated to shear cross-linked chromatin. The protein–DNA complexes were immunoprecipitated using an anti-Osx antibody (Abcam Inc., Cambridge, MA, USA) or immunoglobulin G (IgG), which served as a control. After immunoprecipitation, the formaldehyde crosslinks were reversed, and the DNA was purified and subjected to PCR amplification. The MMP9 promoter fragment (−1242 to −875 bp) containing the CCAAT sequence was amplified using the forward primer 5′-CATGGAGCAGGGCTGGAG-3′ and the reverse primer 5′-CCGCAACGGATGGTGGTG-3′. The PCR products were separated on a 2.0% agarose gel, and the resulting DNA bands were recorded.

### Western blotting

The following primary antibodies were used for immunoblotting: anti-Osx antibody (Abcam); anti-Flag antibody (Sigma Chemical Co., St Louis, MO, USA); anti-MMP13, anti-VEGF, anti-CD34, and anti-PTHrP antibodies (Protech, Nanking Dist, Taipei, Taiwan); and anti-β-actin, anti-rabbit, anti-rat, and anti-mouse IgG antibodies (Bioworld Technology, Minneapolis, MN, USA).

### Zymography

Cells were seeded in six-well tissue culture plates and allowed to adhere in the presence of serum. Then the serum was withdrawn for 24 h. The medium was centrifuged to remove cellular debris. Concentrated samples with equal amounts of proteins were mixed with SDS sample buffer without reducing agent and subjected to 8% SDS–PAGE containing 0.1% gelatin A (Sigma). After electrophoresis, the gels were washed several times in 2.5% Triton X-100 for 1 h at room temperature to remove the SDS, and then incubated for 24–48 h at 37 °C in buffer containing 5 mM CaCl_2_ and 1 mM ZnCl_2_. Thereafter, the gels were fixed and stained with 0.25% Coomassie Blue R-250 for 4 h, and then destained in 45% methanol and 10% acetic acid. The molecular weights were estimated by reference to prestained SDS–PAGE markers.

### Quantitative RT-PCR (qRT-PCR)

From the indicated cells, total RNA was isolated with RNAiso Plus Reagent, and cDNA was synthesized with a PrimeScript RT Reagent Kit, according to the manufacturer′s protocol. Quantitative PCR was performed with FastStart Universal SYBR Green Master Mix (Rox) using a Roche LightCycler^®^ 96 Real-time PCR System. The following primers were used for the experiment: hMMP9 F: 5′-TGACAGCGACAAGAAGTG-3′ and hMMP9 R: 5′-CAGTGAAGCGGTACATAGG-3′; hMMP13 F: 5′-AAATTATGGAGGAGATGCCCATT-3′ and hMMP13 R: 5′-TCCTTGGAGTGGTCAAGACCTAA-3′; hIL-8 F: 5′-CTGCGCCAACACAGAAATTAT-3′ and hIL-8 R: 5′-TTCACTGGCATCTTCACTGATTCTT-3′; hVEGF F: 5′-CAGAATCATCACGAAGTG-3′ and hVEGF R: 5′-TCTGCATGGTGATGTTGGAC-3′; and hPTHrP F: 5′-CTGGTTCAGCAGTGGAGC-3′ and hPTHrP R: 5′-TTCTGCGATCAGATGGTG-3′. β-Actin, which served as an internal control, was amplified simultaneously with the following primers: β-actin F: 5′-AGATGTGGTCAGCAAGCAG-3′ and β-actin R: 5′-GCGCAAGTTAGGTTTTGTCA-3′. The relative expression levels of the target genes were normalized to those of β-actin and were calculated using the 2^−ΔΔCt^method.

### Measurement of secreted factors

For MDA-MB-231 cell supernatants or tumor tissue slurries, IL-8 and PTHrP levels were determined with ELISA kits (Huijia, China).

### Animal model construction

All procedures involving mice and the corresponding experimental protocols were approved by the Animal Care and Use Committee of Nanjing Medical University. Briefly, 2.5 × 10^6^ cells were inoculated into the mammary fat pads of 4-week-old female nude mice. Primary tumor growth was measured with calipers every 2 days. Six weeks thereafter, the tumor weights were determined, and the tumors were snap-frozen in liquid nitrogen or placed in 4% paraformaldehyde and stored at −80 °C until analysis. Cells (1 × 10^5^) were injected into the tibiae of 4-week-old female nude mice according to a method described previously^41^. Metastasis development was monitored by radiography and microCT.

### Bone histology and histological analysis

The hind-limb long bones were excised, fixed in 10% neutral-buffered formalin for 24 h, decalcified (10% EDTA, 2 weeks), dehydrated by a graded alcohol series, and embedded in paraffin. Histological sections (5 μm) of decalcified tibiae (in 5 μmol/l EDTA, 4 weeks) were stained with HE using standard protocols. The osteoclasts were detected by tartrate-resistant acidic phosphatase (TRAP) staining.

### Immunohistochemical analysis

After deparaffinazation and rehydration of the sections from tumor samples, antigen retrieval was performed in a pressure cooker. Endogenous peroxidase activity was blocked with 0.5% hydrogen peroxide for 10 min. Antigens were recovered by heating the slides in an autoclave sterilizer for 2 min in 0.01 mol/l Tris–HCl at pH 6.0. The sections were incubated overnight at 4 ℃ with a primary antibody. The sections were then incubated with the secondary antibody for 30 min and then visualized with 3,3′-diaminobenzidine (DAB). Immunohistochemical assays were evaluated by two independent investigators using a semiquantitative scale. The percentages of stained cells were recorded, and each sample was classified according to a specific expression pattern for each antibody and the number of positive cells.

### Ethics statement

All participants provided written informed consent prior to participating in the study. The ethics committee of Outdo Biotech Co, Ltd., Shanghai, approved the study protocol.

### Statistical analysis

Comparisons between Kaplan–Meier curves were performed using the log-rank test. Other comparisons were made using an unpaired two-sided Student’s *t*-test without an assumption of equal variance or a nonparametric Mann–Whitney *t*-est.

## Supplementary information

Supplementary Fig. 1

Supplementary figure legends
