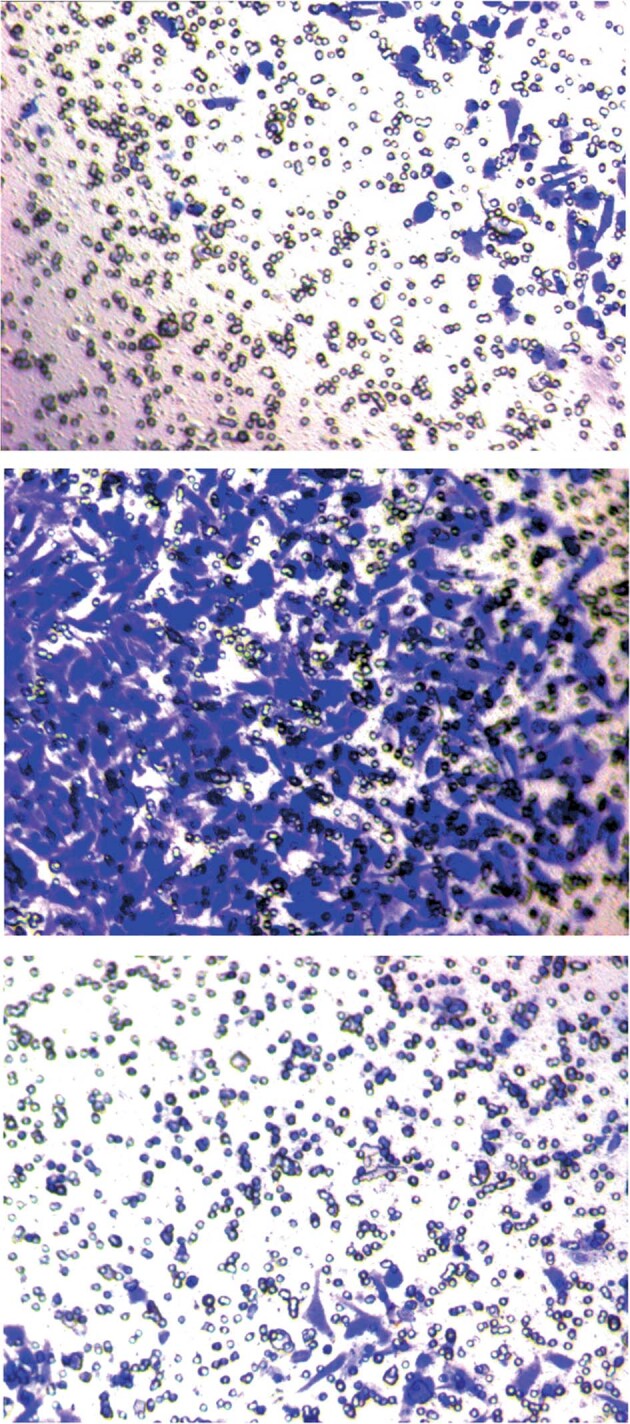# Correction to: Upregulated osterix promotes invasion and bone metastasis and predicts for a poor prognosis in breast cancer

**DOI:** 10.1038/s41419-022-04545-x

**Published:** 2022-02-08

**Authors:** Bing Yao, Jue Wang, Shuang Qu, Yang Liu, Yuci Jin, Jianlei Lu, Qianyi Bao, Lingyun Li, Hongyan Yuan, Changyan Ma

**Affiliations:** 1 Jiangsu Key Laboratory of Xenotransplantation, Nanjing Medical University, Longmian Road 101, 211166 Nanjing, Jiangsu China; 2 Department of Medical Genetics, Nanjing Medical University, Longmian Road 101, 211166 Nanjing, Jiangsu China; 3 Division of Breast Surgery, the First Affiliated Hospital with Nanjing Medical University, 210029 Nanjing, China; 4 Department of Orthopedics, the First Affiliated Hospital with Nanjing Medical University, 210029 Nanjing, China; 5 Department of Oncology and Lombardi Comprehensive Cancer Center, Georgetown University Medical Center, Washington, DC 20007 USA

**Keywords:** Breast cancer, Cell invasion

Correction to: *Cell Death Dis* 10.1038/s41419-018-1269-3, published online 10 January 2019

The original version of this article unfortunately contained an error in Figure 2. The authors apologize for the error. The corrected figure can be found below.