# Supplementary figures and images for: Upregulated osterix promotes invasion and bone metastasis and predicts for a poor prognosis in breast cancer
Source: Cell Death Dis. 2019 Jan 10;10(1):28. doi: 10.1038/s41419-018-1269-3 (PMC6328543; doi:10.1038/s41419-018-1269-3)

**Supplemental Figure 1.**

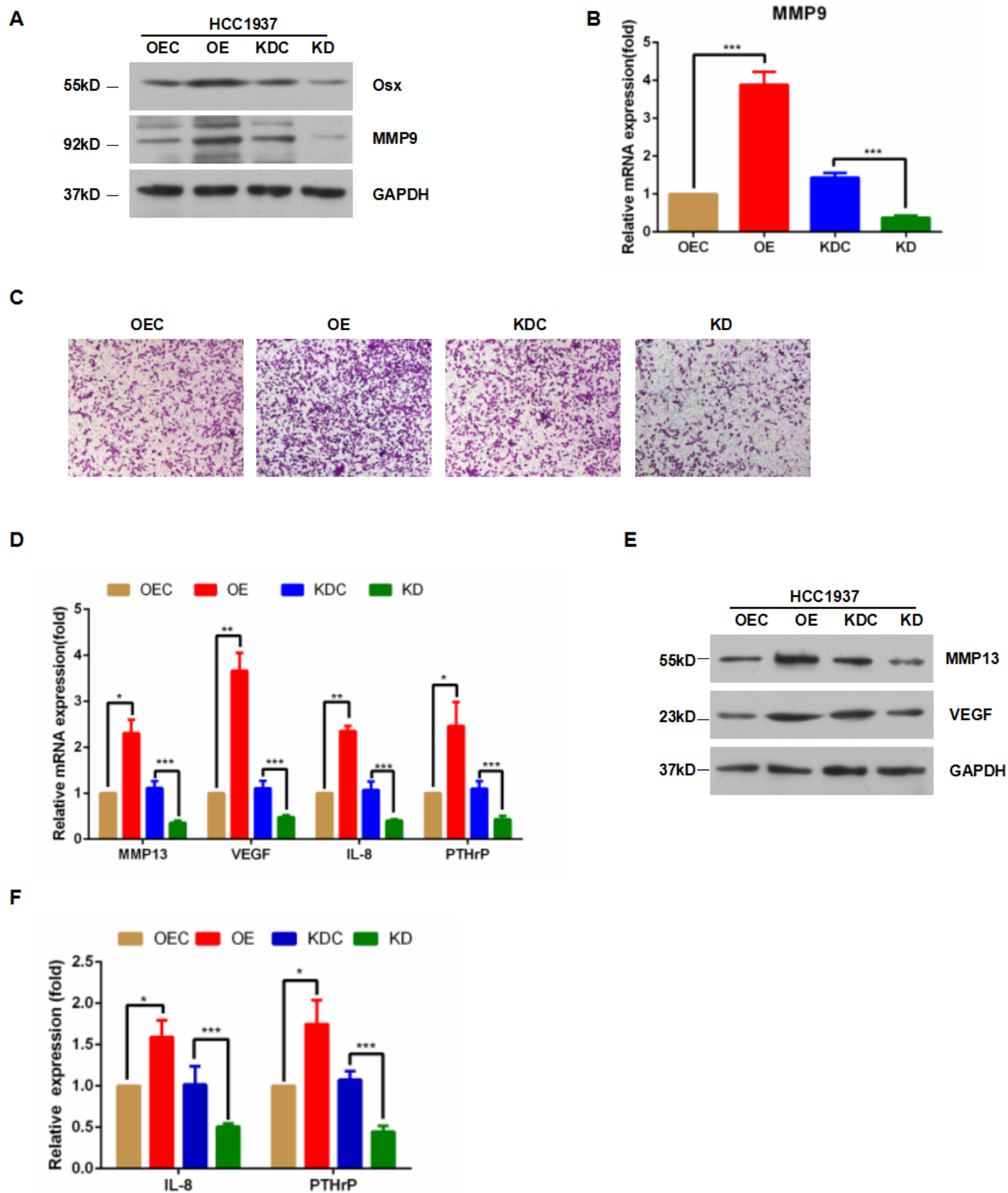

Supplement: Supplementary file 1 — Supplementary Fig. 1 [file 41419_2018_1269_MOESM1_ESM.pdf]
