# Supplementary material for: Upregulated osterix promotes invasion and bone metastasis and predicts for a poor prognosis in breast cancer
Source: Cell Death Dis. 2019 Jan 10;10(1):28. doi: 10.1038/s41419-018-1269-3 (PMC6328543; doi:10.1038/s41419-018-1269-3)
Supplement: Supplementary file 2 — Supplementary figure legends [file 41419_2018_1269_MOESM2_ESM.doc]

**Figure Legends**

**Supplemental Figure 1.** Osx promotes the invasiveness of HCC 1937 cells. HCC 1937 cells were transiently transfected with recombinant plasmids containing Osx cDNA and Osx-specific siRNA, or the corresponding controls. (a) MMP9 protein expression was detected by Western blot analysis. GAPDH was used as an internal control. (b) MMP9 mRNA expression was detected by Real-time PCR, respectively. β-Actin was used as an internal control. (c) Transiently transfected HCC 1937 cells were seeded into Matrigel-coated invasion chambers and incubated for 24 h. Representative images are shown. (d) mRNA expression of MMP13, VEGF, IL-8 and PTHrP in transiently transfected HCC 1937 cells was detected by Real-time PCR. β-Actin was used as an internal control. (e) MMP13 and VEGF protein expression was detected by Western blot analysis. GAPDH was used as an internal control. (f) IL-8 and PTHrP protein levels in culture media from transiently transfected HCC 1937 cells were determined by ELISA. Values are presented as the mean ± SD. * indicates p < 0.05, ** indicates P<0.01, *** indicates P<0.001.
